# A Portable, Patient-Possessed Health Record: Architecture for Care Coordination as an Alternative to Centralized Data Aggregation

**DOI:** 10.3390/pharmacy14040103

**Published:** 2026-07-08

**Authors:** Richard Henry Parrish

**Affiliations:** Alice L. Walton School of Medicine, Bentonville, AR 72712, USA; richard.parrish@alwmed.org; Tel.: +1-(706)-321-7218

**Keywords:** health records, health information exchange, interoperability, medication reconciliation, pharmacy, privacy, prescription drug monitoring programs, surveillance, FHIR, SMART Health Cards

## Abstract

The fragmentation of clinical information across health systems, community pharmacies, and specialty providers continues to undermine medication safety and emergency care, particularly when patients are unconscious or otherwise unable to communicate their history. The dominant response to this fragmentation has been the construction of a centralized data infrastructure—health information exchanges, prescription drug monitoring programs (PDMPs), and federated electronic health record (EHR) networks—that aggregates clinical information into institutional databases that are queryable by providers, insurers, regulators, and, in many jurisdictions, law enforcement. This article argues that the same care-coordination problems can be addressed through an architecturally different approach in which the patient, not the institution, holds the integrative artifact. The proposed design, here labeled the Guardian Card (a conceptual architecture, not a commercial product), pairs an HL7 Fast Healthcare Interoperability Resources (FHIR) clinical payload with the SMART Health Cards verifiable-credential framework and a dual-modality (QR code plus near-field communication) physical carrier. After describing the technical architecture, hardware options, and a five-phase deployment roadmap, the design is situated within the surveillance-critical scholarship that has documented PDMP function creep, third-party doctrine erosion, racial disparities in algorithmic prescribing oversight, and the surveillance-instrumentarian repackaging of nominally de-identified prescription data. The Guardian Card is offered as one operational implementation of a patient-controlled medication-record architecture, with community pharmacy and long-term post-acute care, where the Pharmacist eCare Plan integration is most feasible as a recommended first-deployment venue.

## 1. Introduction

Two distinct problems converge on the design of contemporary patient health records, and most current architectures address only one of them. The first is clinical fragmentation: personal health information remains scattered across EHRs, retail and specialty pharmacies, payer systems, and patient-held paper, producing the medication discrepancies and emergency-department delays that have motivated two decades of personal-health-record advocacy [1,2]. The second, more recently surfaced problem is structural surveillance: the dominant architectures proposed to solve clinical fragmentation, centralized health information exchanges, mandatory PDMP reporting, and federated cross-EHR querying, produce, as a structural side effect, one of the most extensive infrastructures for the population-scale collection of biological and pharmaceutical data in contemporary United States practice [3,4,5].

The two problems are typically treated as separate domains. Care-coordination researchers focus on interoperability standards and clinical outcome metrics, while privacy and constitutional scholars focus on PDMP litigation, HIPAA exceptions, and third-party doctrine reform. This article argues that they should be treated jointly, because architectural choices that address one tend to worsen the other. A clinical information substrate that any authorized provider can query in real time is, by construction, also a substrate that any other authorized actor can query in real time, and the political economy of who counts as authorized has shifted substantially across the four decades of pharmaceutical data infrastructure deployment. PDMPs began as tools for prescribers and have, in nearly half of U.S. states, become databases queryable by law enforcement without a warrant or subpoena [3,4]. The Sixth Circuit recognized the constitutional implications of this shift in *United States v. Martin* (2020), holding that the reasoning in *Carpenter v. United States* (2018) about compelled disclosure to third parties applies to prescription records, but the holding is jurisdictionally limited, and the underlying architectural surveillance capacity remains intact [5,6].

Recent empirical work documents the welfare consequences in populations the surveillance apparatus was nominally designed to protect. Joshi et al. found that must-query PDMP mandates were associated with increased overdose deaths among Black and Hispanic individuals relative to White individuals in the same period [7]. Mandatory PDMP-use policies have been associated with measurable declines in buprenorphine distribution for opioid use disorder treatment [8]. Transgender patients in states where gender-affirming care is politically contested face the prospect of warrantless law enforcement access to their testosterone prescription histories [3]. And the nominally de-identified prescription data that flows outside HIPAA’s jurisdiction is, in practice, frequently re-identifiable through routine record linkage [9], then repackaged for advertising and underwriting in the commercial data economy that Zuboff terms “surveillance instrumentarianism” [10], for “behavioral modification, prediction, monetization, and control” (p. 352).

The structural critique that emerges from this literature has a clean statement: when the underlying intervention that generates the clinical data is government-mandated rather than truly voluntary, the third-party doctrine’s “abandonment” premise does not apply, but the constitutional protection the doctrine withdrew has not been restored [7,8,9]. The PDMP is the canonical instance, but the same logic applies to any architecture in which clinical data flows compulsorily through institutional intermediaries that aggregate, retain, and onward-share it.

This article describes a portable, patient-possessed health record—the Guardian Card—that is intentionally designed to resolve the care-coordination problem without perpetuating a broader data-aggregation footprint. The name “Guardian Card” is used throughout as a convenient label for this conceptual architecture and does not refer to any commercial product, brand, or existing system; to the author’s knowledge, no product with this name is marketed. The architecture rests on three design commitments. First, the integrative artifact lives on the patient’s person, not in an institutional database; the credential itself is the source of truth, signed by an authoritative issuer but stored and presented by the patient. Second, third-party access requires per-event consent rather than prior bulk authorization, modeled on the SMART on FHIR authorization sequence rather than on the implied consent fiction that underwrites most current health-information exchange. Third, the audit trail is visible to the patient, not only to the institutional steward, inverting the asymmetry between institutional and patient visibility that has come to characterize the existing pharmaceutical surveillance apparatus [10,11].

The clinical functions the Guardian Card serves are the same as those served by a tethered personal health record—medication reconciliation at care transitions, emergency access to allergies and active medications, and care coordination within interprofessional teams in which the pharmacist, as a full member, is responsible for optimizing drug therapy—but the data flow is architecturally inverted. Where a centralized health information exchange aggregates patient data into a queryable institutional substrate, the Guardian Card distributes the substrate into millions of patient-held artifacts that institutions can read only with consent. The technologies required to do this now securely exist in production form: HL7 FHIR for data representation [12,13], the World Wide Web Consortium’s Verifiable Credentials Data Model paired with SMART Health Cards for cryptographic portability [14,15], and dual QR/near-field communication carriers for offline reads in emergency settings [16]. What is missing is not the technology but a design discipline that treats the surveillance threat model as a first-class architectural constraint rather than as an afterthought.

The federal government’s engagement with patient-possessed health records extends back to the Markle Foundation’s Connecting for Health initiative, which—with active ONC and federal-agency participation—defined the personally controlled health record in 2003 as one in which the locus of control is the patient [1,2]. The American Health Information Community (AHIC), a federal advisory committee convened by HHS in 2005, identified personal health records as a core Consumer Empowerment use case, and the Department of Veterans Affairs’ Blue Button program, launched in 2010 with a direct presidential announcement, gave veterans a mechanism to download their records as portable files [17]. Despite these initiatives, federal architecture has never made the patient the primary custodian of the authoritative record. The HITECH Act of 2009 directed approximately $35 billion in meaningful use incentive payments to eligible professionals and hospitals—institutional custodians of provider-held electronic health records—rather than to patients holding portable credentials [18], structurally directing the entire infrastructure investment toward institutional data custody. The Nationwide Health Information Network (NHIN), intended to federate exchange across institutions, kept data at the originating providers rather than relocating it to patients. 

When ONC abandoned formal NHIN rulemaking in 2012, the network transitioned to the privately operated eHealth Exchange and the architecture remained provider-to-provider [17]. Electronic health record (EHR) vendors, whose certified platforms became the required substrate for eligibility, maintained proprietary data custody as a structural competitive asset, a practice Congress characterized as information blocking and addressed in the 21st Century Cures Act of 2016 [19]. The cumulative result was that every federal initiative from the AHIC Consumer Empowerment workgroup through the Trusted Exchange Framework and Common Agreement (TEFCA) created patient access to records held by institutions without making the patient the primary custodian of the authoritative record. Patient possession remains the unresolved design problem. That gap is not merely technical. It is a public-choice outcome reflecting the concentrated interests of providers, health systems, and EHR vendors in retaining data custody [5].

## 2. The Centralization Trap: How Care-Coordination Infrastructure Becomes Surveillance Infrastructure

The case for the Guardian Card cannot be made on clinical-utility grounds alone, because the centralized alternatives serve the same clinical functions. The case must be made structurally, by identifying what centralized architectures produce that patient-possessed architectures do not. This section briefly synthesizes the empirical and constitutional literature on that question [20].

### 2.1. The PDMP as the Canonical Centralized Case

Every controlled substance prescription filled in the United States, and in some jurisdictions every prescription regardless of schedule, generates a record reported to a state PDMP, typically within 24 h [21]. All 50 states and the District of Columbia operate such programs; the majority require prescribers to query the program before issuing controlled substance prescriptions, and 48 participate in an interstate data-sharing network through the National Association of Boards of Pharmacy that produces, in practical effect, a near-real-time national database of controlled substance prescribing [21,22]. The infrastructure was assembled incrementally and was justified at each stage by narrow public-health rationales, but its current operational reach extends well beyond those rationales. Federal mandatory electronic prescribing under the SUPPORT for Patients and Communities Act has, since 2023, extended the reporting infrastructure to all Schedule II–V controlled substances under Medicare Part D, and earlier state mandates such as New York Public Health Law § 281 in 2016 had already extended electronic prescribing to all prescription categories regardless of schedule [23]. The same interoperable infrastructure that captures controlled-substance data is technically capable of capturing every prescription category, requiring only the political will to do so [21,24].

What these databases capture extends far beyond a simple record of dispensed medications. The conditions PDMP records document, such as chronic pain, anxiety disorders, opioid dependence, psychiatric illness, and ADHD, carry consequences in employment, professional licensure, child custody, and immigration status that patients rarely anticipate when their prescriber transmits a prescription to a pharmacy. Because the PDMP links the patient to the prescribing clinician, the dispensing pharmacy, the fill frequency, and the cross-provider pattern over time, it assembles a behavioral portrait substantially richer than any single transaction within it, and one whose value for law enforcement investigation or commercial profiling derives from exactly that composite character [4,21].

### 2.2. Function Creep and the Surveillant Assemblage

Surveillance scholarship identifies two structural tendencies that bear directly on the design of any centralized clinical-data architecture. The first is “function creep:” the expansion of a surveillance technology beyond its original purpose through incremental redefinition of permissible use [24]. PDMPs originated as healthcare tools for prescribers, were expanded with national pharmacy board connections, were further opened to law enforcement under state laws explicitly authorizing such access, and now operate in many jurisdictions as criminal-investigation databases [3,4]. The data does not change. Only the political will required to redirect it does.

A second structural tendency operates at a higher level of abstraction. Haggerty and Ericson coined the term “surveillant assemblage” to describe the emergent result when digitization connects formerly separate data systems into shared infrastructure that is greater than the sum of its parts [25]. Isolated streams, a prescription fill here, a laboratory result there, a claims history from a third institution, lose their distinct origins as they flow into common repositories, and what gets put back together at the other end is not simply a reconstructed medical record but a composite portrait assembled from fragments the patient never understood to be continuous. No single architect designed the fusion, and no single authority controls it; the process grows by connecting adjacent infrastructure wherever technical opportunity and institutional incentive converge. A PDMP database integrated with an EHR, tethered to pharmacogenomic profiles and cross-referenced against a forensic genetic database, is this assemblage in its biological expression [25,26], and each new clinical-data system joins it by default unless deliberately architected to remain apart.

### 2.3. Third-Party Doctrine and the Constitutional Gap

The constitutional doctrine that nominally governs government access to clinical data was designed for a transactional era it cannot any longer describe. The third-party doctrine, established in *United States v. Miller* [27] and *Smith v. Maryland* [28], held that individuals lack reasonable expectations of privacy in information voluntarily shared with third parties. Applied to prescription records, the doctrine yields the result that a patient who fills a controlled-substance prescription has “voluntarily shared” that information with the pharmacy and therefore retains no constitutional privacy interest against subsequent government access. *Carpenter v. United States* recognized the inadequacy of this reasoning in the context of cell-site location information, and *United States v. Martin* [5] extended *Carpenter’s* logic to PDMP records, holding that prescription information shared under a state regulatory mandate is not voluntarily shared in the sense the third-party doctrine requires [5,6]. The holdings are important but jurisdictionally limited, and the underlying architectural fact remains. In nearly half of American states, law enforcement can query the PDMP without a warrant or subpoena [3].

Ferguson identifies three structural conflations in the current Fourth Amendment doctrine that prevent courts from recognizing what comprehensive clinical surveillance does at scale. The conflation of privacy from government with privacy in general; the conflation of privacy with secrecy; and the failure to conceptualize collective privacy harms produced by surveillance that operates at population scale rather than against specific individuals [29]. Architectures that aggregate clinical data at the institutional level make all three conflations operationally consequential. Architectures that distribute the integrative artifact to patients neutralize the first two structurally and substantially reduce the surface area of the third.

### 2.4. Welfare Costs Concentrated in Vulnerable Populations

The empirical literature on PDMP welfare effects shows that the costs of centralized prescription surveillance are not distributed evenly. Lagisetty et al. documented buprenorphine treatment disparities by race and payment source [30]. Joshi et al. found that must-query PDMP mandates were associated with increased overdose deaths among Black and Hispanic individuals relative to White individuals during the same period [7]. Townsend et al. reported disproportionate impacts on high-dosage opioid receipt among Black patients [31]. Johnson et al. found that mandatory PDMP-use policies were associated with an 8 percent decrease in buprenorphine distribution for opioid use disorder treatment, a shift toward more restrictive treatment modalities for the patient population least able to absorb that shift [8].

The same pattern recurs in transgender patient populations. McCreedy et al. documented that in many states permitting warrantless PDMP access, testosterone prescription data is accessible to law enforcement without individualized suspicion or judicial approval—information that, in jurisdictions where gender-affirming care is politically contested, creates the prospect of medical records being used adversely to the patients who generated them [3]. Chronic pain patients face a corresponding deterrent: PDMP query mandates have been associated with reductions in opioid prescribing that affect clinically appropriate and inappropriate prescribing simultaneously, leaving patients with undertreated pain whose welfare costs the surveillance system does not measure [32,33]. These distributional effects are not incidental artifacts of imperfect implementation. They are the predicted output of a system whose institutional beneficiaries—regulatory agencies, commercial data aggregators, law enforcement, and the insurance and pharmacy–benefit-management complex—are different from the patient populations whose data populates it, a pattern political scientists and surveillance sociologists have characterized as social sorting [20,34,35].

### 2.5. De-Identification’s Empirical Failure

A common defense of centralized clinical data infrastructure is that downstream commercial uses operate on de-identified data and therefore raise no individual-privacy concern. Sweeney demonstrated empirically that nominally de-identified health data can frequently be re-identified by combining it with other publicly available data sources, particularly for patients with distinctive prescription patterns, rare diagnoses, or unusual demographic combinations [9]. The Federal Trade Commission’s 2023 enforcement action against GoodRx, in which prescription discount data had been repackaged and sold to advertising networks, established that the commercial economy operating on prescription information extends well beyond the covered entities HIPAA governs [36]. The data exits HIPAA’s jurisdiction at the points where it becomes most commercially valuable, and the privacy framework intended to govern it does not reach the actors who profit from it.

Zuboff traces the commercial logic organizing these flows [10]. When prescription claims, pharmacy loyalty histories, coupon-search records, and medication-advertising engagement are combined, the resulting behavioral profile acquires commercial value for pharmaceutical manufacturers, insurers, and advertising networks that is inseparable from its predictive character. The profile is worth purchasing precisely because it allows the buyer to anticipate and shape the decisions of the patient whose data comprises it. Prescription data is not a byproduct of the clinical relationship; in this economy it is the relationship’s primary output, and its journey from pharmacy counter to commercial marketplace passes through the HIPAA exceptions that were never designed to prevent it.

### 2.6. Implications for Portable Health-Record Design

The implications for a portable patient health record are direct. A design that aggregates patient data into an institutional database, however well-intentioned, joins the surveillant assemblage by default. A design that issues a cryptographically signed credential to the patient, requires per-event consent for institutional access, and writes a patient-visible audit log of every read does not eliminate every surveillance concern; the threat model in Section 8 enumerates what it does and does not address. But it inverts the architectural asymmetry that makes the centralized model structurally vulnerable to the function creep, third-party doctrine erosion, and surveillance-instrumentarian repackaging documented above [10]. The remaining sections describe how that inverted architecture can be constructed using existing standards.

### 2.7. International Context and the Recognized Benefits of Centralized Architectures

The structural critique developed above should not be read as a claim that centralized clinical-data architectures lack value. They deliver substantial and well-documented benefits. Aggregated data have enabled large-scale pharmacovigilance, post-market detection of adverse-drug-event signals invisible at the level of the individual encounter, and research that depends on linkage across large cohorts. Centralized exchanges support population-health management and public-health emergency response, reduce duplicative testing, and enable cross-provider interaction checking. The prescription-monitoring infrastructure criticized here has plausibly contributed to reductions in duplicative controlled-substance prescribing in some settings. A balanced appraisal recognizes these gains. The argument of this article is not that aggregation yields no benefit, but that the architecture through which the benefit is obtained also creates a concentrated capacity for secondary use whose governance has not kept pace, and that an alternative architecture can preserve much of the clinical benefit while reducing that concentrated capacity.

International experience situates the proposal within a broader landscape. Several national systems pair centralized records with strong patient-facing governance. Estonia’s national health information system, for example, is widely noted for recording every access to a patient’s data in a log the patient can inspect, illustrating that audit transparency need not be sacrificed to centralization. The European Union’s General Data Protection Regulation establishes explicit-consent and purpose-limitation requirements that constrain secondary use more tightly than the United States sectoral framework [37], and the emerging European Health Data Space contemplates patient-controlled sharing at continental scale. These models differ from the patient-possessed approach proposed here, but they share its core commitment to patient visibility and control, and they indicate that the design space is wider than the centralized-versus-portable dichotomy alone suggests. A full comparative evaluation of national architectures is beyond the scope of this conceptual article. The narrower point is that patient-controlled governance is achievable through more than one architecture, and that the portable credential is one option that is particularly suited to the United States legal context. Table 1 summarizes representative national systems across both architectures, illustrating the range of governance models and the centralized orientation of most established implementations.

## 3. Technical Framework: The SMART Health Cards Standard

### 3.1. HL7 FHIR as the Data Language

To ensure that the card’s clinical payload can be parsed by any modern EHR or pharmacy system, the data should be expressed in HL7 FHIR. FHIR was designed as an agile, RESTful, web-friendly alternative to earlier HL7 specifications, modeling clinical content as discrete resources—Patient, MedicationStatement, AllergyIntolerance, Immunization, Condition, and so on—that can be requested and exchanged over standard HTTP [12]. The SMART on FHIR specification layers a standards-based application model on top of FHIR, including an OAuth 2.0 authorization sequence that allows third-party applications to be written once and launched across compliant EHRs [13]. For a portable record, the practical implication is that the credential’s contents can be expressed as a FHIR Bundle containing the most safety-critical resources, with the bundle then wrapped as a verifiable credential.

### 3.2. Data Minimization Through Verifiable Credentials

A complete clinical record cannot fit inside a QR code. Even with aggressive compression, the SMART Health Cards specification practically limits payload size to a handful of FHIRs [15]. This constraint is a structural feature rather than a limitation to be engineered around. The World Wide Web Consortium’s Verifiable Credentials Data Model is explicitly designed around selective disclosure. A credential expresses a minimum-necessary set of claims, signed by the issuer, that a verifier can validate without contacting the issuer or any central registry [49]. When applied to a portable health record, this means the card itself carries only the data a first-responder or community pharmacist needs at the point of contact, with deeper records reachable through a separately authorized channel.

Data minimization has a secondary virtue that is essential to the surveillance threat model. The re-identification risks Sweeney documented for nominally de-identified clinical data scale with the dimensionality and granularity of the data record [9]. A credential constrained to a small number of safety-critical FHIRs presents a substantially smaller re-identification surface than a comprehensive longitudinal record, even in the limited case in which the credential was somehow obtained by an adversary. The same constraint that protects against re-identification also limits the commercial-aggregation value of any single credential, structurally undermining the surveillance-instrumentarian incentive to compromise the system [10].

### 3.3. Two-Tier Access Architecture

Operationally, the credential is partitioned into two access tiers. The first tier, a locally readable “Medical ID” layer, contains a small, unencrypted or symmetric-key-protected subset of safety-critical data: name and date of birth, blood type, drug allergies, an active medication list, primary diagnoses, advance-directive status, and emergency contacts. This tier is scannable by any smartphone camera using the standard SMART Health Cards verifier pattern, supporting first-responder use cases without requiring patient consent or network connectivity. The second tier, a consent-gated layer, embeds a deep link in the same QR or near-field communication payload that resolves through a secure gateway to the patient’s full longitudinal record. Authorization for the second tier is mediated through a real-time consent prompt to the patient’s mobile device, modeled on the SMART on FHIR OAuth 2.0 launch flow [13].

This split is more than an engineering convenience. It is the operational implementation of the constitutional distinction between compelled and voluntary disclosure that post-Carpenter doctrine, traced through *United States v. Martin*, identifies as the constitutional fault line in current clinical-data governance [4,5,6]. Tier 1 access is justified by the same emergency-exception reasoning that has always governed first-responder action. It is bounded by the minimum-necessary principle and by the structural impossibility of using the Tier 1 payload as a substrate for aggregate surveillance. Tier 2 access requires affirmative, contemporaneous, per-event consent from the patient—not the legal-fiction “consent” constructed by a notice provided at intake and forgotten by the patient before the data leaves the institution’s control.

## 4. Hardware Implementation Options

### 4.1. The “Smart” Credit Card (NFC + QR)

A wallet-sized PVC card offers a tactile, low-cost carrier familiar to patients and clinicians alike. The front surface carries printed identification (name, photograph, date of birth, blood type) alongside a high-contrast QR code, while an embedded passive near-field communication (NFC) chip provides a separate, tap-to-read channel. The two modalities are complementary rather than redundant. QR codes are universally readable by any smartphone camera without specialized hardware—the property that made SMART Health Cards deployable at the population scale during the COVID-19 response [14]—but they can be photographed surreptitiously at a distance. NFC, by contrast, requires approximately two inches, which substantially reduces casual surveillance risk. A simulation study in a hospital emergency department found that NFC-mediated patient lookup on a mobile EHR reduced physician turnaround time compared with conventional desktop workflows, suggesting that the tap-to-access pattern is well suited to emergency department conditions [16].

Physical possession of the credential carries an additional, often overlooked constitutional benefit. A credential physically held by the patient is, in the Fourth Amendment’s traditional categories, among the patient’s “papers and effects,” and its compelled production by the government is governed by the warrant requirement in ways that institutional records typically are not [29]. The architectural choice to make the credential a physical artifact carried by the patient is, in this sense, a constitutional design choice as much as a clinical-usability one.

### 4.2. The Durable QR Keychain or Wristband

For pediatric and geriatric populations, a wallet card is easily lost or separated from the patient. A silicone wristband or rugged keychain fob bearing a laser-etched QR code is substantially more resilient and remains physically associated with the patient through ambulance transport and emergency department triage. The trade-off is reduced data capacity—a single QR rather than QR plus NFC—and somewhat lower legibility once weathered, both of which can be partially mitigated by encoding the QR at a high error-correction level. For patient populations particularly exposed to surveillance harm—chronic pain patients in restrictive jurisdictions, transgender patients receiving testosterone, and individuals receiving treatment for opioid use disorder—the wristband form factor has the additional advantage of being readily removable when the patient is in environments where the credential’s contents might be coerced rather than consented to.

### 4.3. Comparison of Access Technologies

Table 2 summarizes the trade-offs between the two access modalities considered for the Guardian Card. Neither modality dominates the other on every dimension, which motivates the hybrid design described in Section 6.

## 5. Deployment Roadmap: A Five-Phase Approach

### 5.1. Phase 1—Data Aggregation

The back end aggregates clinical information from the patient’s providers using FHIR APIs aligned with the United States Core Data for Interoperability (USCDI) profile [13]. The aggregation is performed under the patient’s authorization, with the resulting bundle prepared for issuance into a credential that is then transferred to the patient’s control. This is the structurally distinguishing feature of the Guardian Card approach. Aggregation occurs but produces a patient-held credential rather than a persistent institutional repository. The pharmacy or health system performing the aggregation retains its own records as required by professional and regulatory obligation, but the integrated cross-source view exists only on the patient’s card.

For pharmacy-originated content, the HL7/National Council for Prescription Drug Programs Pharmacist eCare Plan (PeCP) provides a FHIR-conformant representation of medication-related care plans, drug therapy problems, interventions, and goals—the pharmacist’s structured contribution to an interprofessional record, originally developed under a C-CDA template and since re-expressed in FHIR [50]. Effective team-based care requires that each member’s clinical work product be legible to other members. The PeCP–EHR integration documented in a Tennessee proof-of-concept study demonstrates that pharmacist-generated FHIRs can traverse directly into the medical office’s EHR without manual transcription, making the pharmacist’s drug-therapy assessments available to the broader care team in real-time [50,51]. For long-term post-acute care, converting pharmacist consultation notes from C-CDA to FHIR should be a core component of the aggregated bundle, so that the medication list on the card reflects a team-reconciled record in which the pharmacist’s drug therapy optimization work is preserved alongside the contributions of the prescribing clinician and other team members.

### 5.2. Phase 2—Generating the Digital Signature

The card’s clinical payload must be cryptographically signed by a trusted issuer—a hospital, health system, pharmacy chain, or accredited platform—using the JSON Web Signature scheme defined by the SMART Health Cards framework [15]. The signature, anchored to a publicly resolvable JSON Web Key Set, allows any verifier to confirm that the credential was issued by a recognized authority and has not been altered, without requiring prior coordination between the verifier and the issuer [49]. This is the property that prevents patients or third parties from forging prescriptions, immunization records, or allergy lists. It is the foundation of the trust model that supported state-level SMART Health Cards deployment during the COVID-19 response [14].

The architectural significance of the signature model extends beyond authentication. Because verification requires only public key material rather than a query to a central registry, the verifier learns nothing about the patient that the credential itself does not disclose, and the issuer learns nothing about when, where, or by whom the credential is verified. This absence of an issuer-side verification log denies the surveillant assemblage one of its most important integration substrates [25]. A signature scheme that required the verifier to call back to a central issuer endpoint would reintroduce the centralized log that the patient-held architecture is intended to eliminate.

### 5.3. Phase 3—The Privacy Gate

Scanning the card does not automatically expose the patient’s full record. A second-tier scan triggers a consent flow modeled on the SMART on FHIR OAuth 2.0 launch sequence [13]. The provider’s application sends an access request that the patient receives as a push notification on a mobile device—for example, a request from a named clinician at a named hospital to view the active medication list—which the patient can approve or deny in real time. Every scan, whether approved or denied, is written to an immutable access log visible to the patient.

The privacy gate is the design element most directly responsive to the third-party doctrine erosion that motivates the surveillance critique. Where the PDMP model treats the act of filling a prescription as constructive consent to subsequent institutional and law enforcement access, the privacy gate restores affirmative, per-event consent as the structural prerequisite for access beyond the emergency tier. The patient who scans her own card to share her medication list with a new specialist has consented in the substantive sense that the third-party doctrine assumed but never operationally required. The patient who declines a scan request has refused consent in a way the architecture, not merely the policy, enforces [5,6].

The patient-visible audit log inverts an asymmetry that has come to characterize the existing pharmaceutical surveillance system: institutional actors see the patient’s data, but the patient does not see who has seen it, nor the algorithmic logic by which institutional actors evaluate her [10,11]. Under the Guardian Card model, every access event, including denied requests, appears in a log the patient can review. This is not an incidental usability feature. It is the mechanism through which the architectural commitment to patient sovereignty becomes empirically observable to the patient herself.

### 5.4. Phase 4—Physical Production

Production-quality cards should be printed on PVC stock with a UV-stable laminate to protect the QR code from fading over the card’s service life. NFC inlays should be embedded between laminate layers to resist mechanical wear. A scratch-off or printed personal identification number, required as a second factor after scanning, provides a simple defense against opportunistic theft for first-tier reads, a pattern long established in patient-identification practice [16]. Replacement workflows should mirror those of credit-card issuers, including remote revocation of the signing key associated with a lost or compromised card. The revocation infrastructure should be designed so that revocation events do not themselves generate a centralized log of patient activity beyond what is strictly required for verifier-side trust decisions.

### 5.5. Phase 5—Emergency “Break-Glass” Protocol

Even with consent-mediated access, the architecture must provide a path for life-and-limb emergencies in which the patient is unconscious and cannot approve a second-tier request. The concept of break-the-glass access is well established in health informatics: it permits a clinician to override normal access controls in true emergencies, subject to mandatory justification, automatic audit logging, and post hoc review [52]. For the Guardian Card, scanning the QR code surfaces a clearly labeled “Break Glass” option visible to the responding clinician. Activating it (a) notifies the patient’s designated emergency contacts in real time, (b) releases only the minimum-necessary dataset—typically allergies, current medications such as anticoagulants, advance-directive status, and emergency contacts—and (c) creates an irreversible audit entry tied to the responder’s credentials.

Break-glass is the design phase where the surveillance threat model reasserts itself most forcefully, and the policy framework around it must be designed accordingly. Break-glass logs are themselves clinical records and, in the absence of explicit constitutional protection, become subject to the same warrantless-access regimes the rest of this article argues against. The recommended policy posture is that break-glass logs are retained for the minimum period required for clinical and quality-assurance purposes, that they are encrypted at rest with keys held by the patient or a patient-designated fiduciary, and that any law enforcement access to break-glass logs requires a warrant supported by individualized probable cause—the same constitutional standard that *Carpenter v. United States* and *United States v. Martin* extend to compelled clinical disclosure [5,6]. Without this policy layer, break-glass becomes the channel through which centralized access could reenter by exception.

### 5.6. Operational Procedures: Loss, Replacement, and Update Cadence

Three operational questions determine whether the architecture is practical at scale: what happens when a card is lost, how a card is replaced, and how a card stays current as the patient’s medications change. Each has a concrete answer. On loss, the signing key associated with a lost or stolen card is revoked at the issuing institution, rendering the missing credential unverifiable. Because verification depends on the issuer’s published key material rather than on a central registry of patients, revocation neither requires nor creates a roster of cardholders. A reasonable service target is revocation within minutes of a patient report through a 24 h issuer line, with a single-use paper credential that is available immediately as an interim measure. On replacement, the workflow mirrors established credit-card practice: identity is re-verified at the issuing pharmacy or health system, a fresh credential is generated from the current source record, and a replacement card is issued, typically same-day on-site or by mail within several business days. On currency, because the Tier 1 payload is a static snapshot, a clinically meaningful medication change should trigger reissuance. In the interim, a Tier 2 lookup resolves to the live source of truth, and scanning clinicians should see the issuance date of the static payload together with a prompt to confirm the medication list against the live record once the snapshot exceeds a defined age, for example, 30 days. These procedures are deliberately modeled on systems that patients and institutions already operate, which lowers the implementation burden and shortens the path to adoption.

## 6. Recommended Hybrid Design: The Guardian Card

Bringing these elements together yields one concrete instantiation of the patient-controlled architecture, here labeled the Guardian Card, in which the printed QR code serves as the “key.” It points to a verifiable credential and a consent-gated deep link while the embedded NFC chip serves as a “secure vault” carrying an offline-readable copy of the highest-priority data points, including drug allergies, active medications (with particular attention to anticoagulants, insulin, antiepileptics, and opioids), advance-directive status, and emergency contacts. The card thus functions in three distinct modes. As a first-responder tool, it offers first-tier and break-glass access without requiring patient consent. As a routine care-coordination tool scanned in a pharmacy or specialist’s office, it offers second-tier access via patient consent. And as a personal record, it is reviewable and updateable by the patient through a companion mobile application.

Structurally, the Guardian Card is the operational expression of a patient-controlled medication-record architecture offered as the appropriate replacement for centralized PDMP infrastructure [20]. A patient liberty-centered commitment—that individuals own their bodies and the data those bodies produce [53,54]—is implemented architecturally rather than merely asserted normatively. Care coordination is preserved because the patient can grant any provider access to any subset of the credential at any time. The surveillance substrate is prevented because there is no institutional database to query in bulk, no central registry to subpoena, and no aggregate that can be repackaged for commercial use. The constitutional remedy the surveillance critique calls for becomes, in the Guardian Card, a deployable technical artifact.

Community pharmacy is a particularly strong venue for initial deployment, for reasons rooted in both team-based care science and surveillance equity. Within an interprofessional care model, the pharmacist’s defined role is optimizing drug therapy—identifying drug therapy problems, resolving discrepancies, and ensuring that the medication regimen is safe, effective, and consistent with the patient’s goals across providers [51,55]. The Guardian Card formalizes that contribution: rather than confining the pharmacist’s drug-therapy assessment to the dispensing record, it packages that work product as an FHIR-conformant credential the entire team can read at any point of care. A Guardian Card issued and maintained through the dispensing pharmacy, rather than from an inpatient EHR, carries the pharmacist’s medication-optimization work product as a first-class data element, complementing the medical record rather than subordinate to it, and the clinical value of that integration has been demonstrated at care transitions specifically [56,57]. The deployment venue also matters for the surveillance critique’s most vulnerable populations: patients with chronic pain, opioid use disorder, gender-affirming testosterone therapy, and behavioral health conditions for whom centralized PDMP access has produced documented welfare harms [3,7,8] are precisely the populations who interact most frequently with community pharmacy. A team-issued, patient-held credential serving these populations directly addresses the welfare costs the centralized model produces.

Table 3 summarizes the proposed architecture in a strengths, weaknesses, opportunities, and threats (SWOT) framework, intended as a compact synthesis for the reader.

## 7. Equity Design Considerations

The structural critique of centralized clinical-data architecture rests substantially on the empirical finding that the welfare costs of that architecture fall most heavily on populations the architecture was nominally designed to protect [3,7,32]. A patient-possessed alternative inherits the responsibility to do better, not the privilege of doing the same. This section catalogs the equity questions a deployed Guardian Card program must answer before its structural advantages translate into welfare gains for the populations most exposed to centralized surveillance harm.

### 7.1. The Populations Most Burdened by Centralized Surveillance Are Those Most Underserved by Centralized Care Coordination

The empirical literature surveyed in Section 2.4 identifies the populations whose interaction with centralized PDMP architecture has produced documented welfare harm: Black and Hispanic patients [7,30,31], patients with opioid use disorder for whom buprenorphine access has narrowed under mandatory-query policies [8,58], transgender patients in jurisdictions permitting warrantless PDMP access [3], and chronic pain patients whose clinically appropriate prescriptions have been reduced alongside inappropriate ones [32,33]. These are not different populations from those underserved by fragmented care coordination; they are largely the same populations, who experience the worst of both architectures simultaneously.

The implication of the design is that the Guardian Card’s value to these patients depends on whether issuance reaches them first, not last. A program issued primarily through academic medical centers and tertiary care systems risks reproducing the access disparities of existing personal-health-record adoption [1,2]. Issuance through interprofessional teams anchored at community pharmacy—where patients with chronic pain, opioid use disorder, and ongoing behavioral health treatment interact most frequently with the clinical system—places the credential at the touchpoint that already serves these populations and where the pharmacist’s drug-therapy optimization role within the team is most directly applicable [50,51,59]. The deployment recommendation in Section 6 is therefore not merely a clinical-feasibility argument; it is an equity argument.

### 7.2. Algorithmic Opacity and a Patient Right to Know What an Algorithm Concluded

A patient-controlled credential addresses surveillance asymmetry at the level of data access. It does not, by itself, address the asymmetry that arises from algorithmic decision-making based on the data once accessed. Obermeyer et al. documented that an algorithm used to manage population health for approximately 200 million patients exhibited racial bias, systematically underestimating disease severity in Black patients and recommending lower levels of care intervention for equivalent clinical need [60]. Comparable findings have followed in the prescription-oversight literature: Oliva on dosing discrimination via PDMP risk scores, Pozzi on epistemic injustice in automated opioid risk scoring, Wang et al. on algorithmic opacity in opioid risk regulation, and Bousman and Eyre on the proprietary character of pharmacogenetic decision-support tools [11,61,62,63].

The Guardian Card’s patient-visible audit log can be extended to address this asymmetry, but only if the architectural commitment is made explicit. Where a Tier 2 access event is followed by an algorithmic recommendation or risk-score assignment, the audit log should record not only the access itself but the algorithmic basis for any decision that follows, in a form intelligible to the patient. This is not a feature the underlying SMART Health Cards specification provides. It is an additional layer the deployment must add, ideally backed by regulatory requirement. Without it, the architecture’s commitment to transparency stops at the institutional doorway through which the data was requested.

### 7.3. Digital Access, Connectivity, and the Paper-Only Fallback

The Guardian Card depends on patient mobile-device access at two points: the initial setup, in which the patient receives and registers her credential, and the per-event consent flow for Tier 2 access. The COVID-19 SMART Health Cards deployment in California documented that uptake was disproportionately concentrated in higher-resource areas [14], and smartphone ownership gaps by age, income, and rural geography are well established in the broader digital-health-equity literature. The risk is particularly acute for the surveillance-vulnerable populations identified above: a chronic-pain patient on Medicaid, an unhoused individual receiving methadone treatment, or a transgender patient relying on a community pharmacy in a restrictive state may have intermittent smartphone access at best.

Two architectural commitments mitigate this risk. First, Tier 1 emergency access works entirely offline by design, so a patient without connectivity is not excluded from the principal clinical use case. Second, the Tier 2 consent flow must include non-smartphone alternatives: SMS-based one-time authorization codes, in-person pharmacist-mediated authorization at the issuing pharmacy, and paper printouts of single-use consent codes that the patient can hand to a clinician on request. The paper-only fallback is not an accessibility nicety. It is the architectural commitment that distinguishes a portable-record program that reaches the surveillance-vulnerable from one that compounds their digital exclusion.

### 7.4. Coerced Consent in Intimate-Partner and Institutional Contexts

Patient sovereignty in name can be coercion in practice. A partner holding the patient’s phone can approve Tier 2 scans on the patient’s behalf; an employer can condition employment on a Tier 2 scan; an insurer or landlord can extract “consent” in a form indistinguishable from voluntary agreement. The threat model in Section 8 acknowledges that coerced consent is the architectural problem the Guardian Card does not solve on its own. Several mitigations are available, however, and the absence of any of them weakens the equity case for the design.

First, second-factor authentication on Tier 2 consent—biometric where possible, otherwise a PIN distinct from any device-unlock PIN—defeats simple phone-holding coercion, though not duress when authentication occurs. Second, a patient-activatable “private mode,” in which the device temporarily suppresses Tier 2 consent prompts entirely and defeats prolonged coercion at the cost of routine care-coordination access during the private window. Third, per-purpose request labels that the requesting entity cannot disguise allow the patient to refuse because of what is being requested and for what reason. Fourth, legal anti-coercion rules restricting which entities may request Tier 2 scans for which purposes—modeled on the European Union General Data Protection Regulation’s explicit-consent and purpose-limitation provisions—are required to prevent the architecture from being weaponized through entirely lawful market pressure. Architectural commitments require legal commitments to be operationally meaningful for vulnerable patients.

### 7.5. Proxy Access for Pediatric, Geriatric, and Cognitively Impaired Patients

The architecture, as described in earlier sections, implicitly assumes the patient is able to manage their own credentials, which is true for most cases but not for all of them. Pediatric patients require parental or guardian access with explicit transitions of authority as the patient ages into competence. Geriatric patients with cognitive impairment require representative access under durable power of attorney for healthcare. Patients with episodic cognitive impairment such as acute psychosis, intoxication, or postoperative confusion require fallback representative access that activates only under specified conditions. HIPAA’s personal-representative framework already governs these cases for traditional records. The Guardian Card’s authorization model requires FHIR-level support for representative roles with explicit legal-authority logging, so that the audit log records not just which credential was used but under which authority basis.

Carceral populations require separate treatment. Incarcerated patients have de facto no privacy from institutional staff, regardless of architecture, and the Guardian Card cannot remedy this directly. What it can do is ensure that the patient’s clinical record, especially medication regimens, allergies, and behavioral-health treatments, follows the patient across facilities, into and out of incarceration, and into community pharmacy on release. For a population whose care continuity is among the worst in the United States health system, this is a meaningful equity contribution even where the surveillance reduction the architecture promises is largely unavailable to the patient during the incarceration period itself.

### 7.6. Trust, History, and the Community-Pharmacy Issuance Pathway

Populations harmed by past medical research and by past and present surveillance of intimate biological information (the Tuskegee survivors and their descendants, the survivors of Indian Health Service sterilizations, transgender patients with rational fear of surveillance in restrictive states, undocumented patients with rational fear of immigration enforcement) have grounds for distrust of any EHR, including one stored on a card in their wallet. The patient-held architecture is more compatible with this distrust than centralized architecture is but does not dissolve it. Trust is at least partly mediated by the institution that issues the credential and the relationship the patient has with that institution.

Community pharmacy, particularly in independent and minority-owned settings, is for many low-income, rural, and minority patients the most accessible and most ongoing point of clinical contact [59]. Pharmacists are among the most frequently consulted health professionals in the United States and, in well-functioning interprofessional teams, are the member most directly responsible for drug-therapy optimization across the patient’s entire regimen [51,55]. The PeCP–EHR integration documented by Hohmeier et al. provides a technically feasible pathway for team-coordinated credential issuance anchored at the community pharmacy [50]. The equity case and the technical-feasibility case converge. Anchoring Guardian Card issuance at the trusted community-pharmacy touchpoint is consistent with where surveillance-vulnerable populations interact with the clinical system, what they already trust within it, and where the standards infrastructure to support patient-controlled credentials already exists.

## 8. Threat Model: What This Architecture Resists, and What It Does Not

A surveillance-aware design is only useful if its scope is honestly stated. The Guardian Card architecture intends to resist a specific set of structural threats and is not intended to address all the concerns that motivate the broader surveillance critique.

### 8.1. Threats the Architecture Resists

The architecture resists bulk institutional aggregation by construction. There is no central database of patient credentials, no institutional registry of who holds a Guardian Card, and no required call-back to an issuer endpoint at verification time. The surveillant assemblage requires integration substrates [25]; the Guardian Card denies it one.

The architecture resists function creep in the operational sense Lyon identifies [24]. PDMP function creep is enabled by the fact that the database already exists and contains the data of interest to whichever institutional actor next establishes legal or political access. A patient-held credential cannot be repurposed in this way because each access event requires fresh patient consent. The political will to redirect the database encounters an architectural floor it cannot dissolve.

The architecture resists warrantless centralized law enforcement queries because there is no centralized location at which to direct such queries. Law enforcement, when seeking a Guardian Card’s contents, must either obtain it from the patient herself—subject to the constitutional protections that govern compelled production of papers and effects—or seek the underlying records held by the issuing institution under existing law, which is no worse than the pre-Guardian Card baseline.

The architecture substantially reduces the re-identification surface area Sweeney documented [9]. Tier 1 payloads are constrained to a small number of safety-critical data points. Tier 2 access produces records that exist only ephemerally in the requesting provider’s session, not as new entries in a downstream aggregation pipeline.

Finally, the architecture undercuts the commercial logic Zuboff identifies as surveillance instrumentarianism [10]. The commercial value of behavioral profiles depends on the ability to aggregate transactional data across millions of individuals into a single queryable substrate. A distributed architecture of patient-held credentials does not yield such a substrate even in the limit case in which individual cards are compromised.

### 8.2. Threats the Architecture Does Not Resist on Its Own

The Guardian Card does not eliminate the prescription requirement itself. The PDMP infrastructure exists because the prescription requirement makes it institutionally necessary; the structural remedy ultimately requires reconsideration of the prescription mandate as well [20], and the Guardian Card is not a substitute for that constitutional argument.

The Guardian Card does not prevent lawfully obtained access. A patient can be required to produce her card by a court of competent jurisdiction acting on a warrant supported by individualized probable cause, and the architecture is explicitly designed to leave that constitutional pathway intact. The architecture’s goal is to require warrants, not to prevent them.

The Guardian Card does not prevent coerced consent. An insurer, employer, or pharmacy benefit manager that conditions a benefit on a Tier 2 scan can extract “consent” in a form indistinguishable from voluntary agreement. Architectural commitments require legal commitment, strong anti-coercion rules limiting which entities may request scans for which purposes, to be operationally meaningful. The European Union’s General Data Protection Regulation provides one model for such anti-coercion rules; in the United States, the legal substrate remains underdeveloped.

Finally, the Guardian Card does not address the underlying institutional incentives that produce surveillance infrastructure in the first place. Buchanan and Tullock’s public-choice analysis predicts that surveillance systems will be captured by concentrated institutional interests as a structural matter, and no technical artifact alone can resist this dynamic indefinitely [34]. The Guardian Card is intended as a useful structural improvement, not as a substitute for the constitutional and political work the surveillance critique calls for.

## 9. Ethical Analysis

Because the proposal is normative as well as technical, it is useful to examine it through the four principles of biomedical ethics—respect for autonomy, beneficence, non-maleficence, and justice [64]. The principlist frame clarifies the design’s aims and simultaneously exposes the tensions a deployed program would have to manage.

### 9.1. Respect for Autonomy

The architecture’s central commitment is to autonomy, understood as effective control. The patient holds the credential, authorizes each disclosure beyond the emergency tier, and can review who has accessed it. This is a stronger operationalization of autonomy than the notice-and-acknowledgment consent that governs most current HIPAA exchange, in which authorization is given once and effectively cannot be withdrawn. The limit, examined in Section 7.4, is that control can be coerced. Autonomy on paper becomes autonomy in fact only where accompanying legal protections constrain who may demand a disclosure.

### 9.2. Beneficence and Non-Maleficence

Beneficence is served by the same clinical functions a tethered record provides: reconciliation at transitions, emergency access to allergies and active medications, and team-based coordination in which the pharmacist’s drug-therapy work is legible to the team. Non-maleficence requires attention to harms the design itself can introduce: a static payload that misleads if stale, an emergency tier that releases data without consent, and a break-glass path that can be abused. The mitigations described above—issuance-date prompts, minimum-necessary emergency payloads, and audited, warrant-gated break-glass logs—are non-maleficence requirements rather than optional refinements. Honest accounting also acknowledges that the centralized systems this architecture would partly displace themselves produce benefits, so the relevant comparison is between two imperfect designs rather than between a flawed status quo and a costless alternative.

### 9.3. Justice

Justice is the principle on which the proposal makes its strongest claim and bears its heaviest burden. The empirical record shows that the costs of centralized prescription monitoring fall disproportionately on Black, Hispanic, transgender, and chronic-pain populations, and a patient-controlled alternative is justified partly as a corrective. But the same distributive concern cuts the other way: a design dependent on smartphones and institutional issuance can reproduce the disparities it seeks to remedy unless issuance reaches underserved populations first and non-digital fallbacks are guaranteed. Justice therefore requires the equity commitments cataloged in Section 7 to be treated as conditions of the design rather than as enhancements to it.

## 10. Limitations and Future Directions

Several issues warrant further work before scaled deployment. First, although the SMART Health Cards framework has been widely adopted in practice, it is not yet an officially adopted standard outside its original vaccination-record use cases. FHIR profiles for broader clinical content, particularly long medication lists, problem lists, and advance directives, require balloting and alignment with current USCDI versions [14].

Second, the QR/NFC carrier is a static object. Each update to a patient’s medications or problem list requires either reissuance of the credential or a second-tier lookup to the live source of truth, raising operational questions about how frequently cards should be re-pressed and how stale-data warnings should be surfaced to scanning clinicians.

Third, digital health equity must be addressed explicitly. The COVID-19 SMART Health Cards deployment in California was used disproportionately by patients in higher-resourced areas, suggesting that programs depending on smartphone-based consent flows risk excluding patients without reliable mobile access [14]. The risk is particularly acute given the surveillance-critical framing of this article: the populations most exposed to surveillance harm under the centralized model may also be the populations least equipped to manage smartphone-based consent. A paper-only fallback consisting of a printed QR code and personal identification number, requiring no application on the patient’s side, is therefore not an optional accessibility feature but a core equity requirement of the architecture.

Fourth, HIPAA’s minimum-necessary standard nominally aligns with the architecture’s commitments but contains exceptions for treatment, payment, health care operations, research, public health activities, and law enforcement under conditions broad enough that a centralized aggregation entity holding even a subset of Guardian Card contents would face HIPAA-permitted disclosure obligations the patient-held architecture is intended to avoid [4,23]. The architecture’s commitments require not just compliance with HIPAA as written but a legal posture in which Guardian Card content is treated as patient property under non-covered-entity rules. The General Data Protection Regulation’s data-minimization, purpose-limitation, and explicit-consent requirements may provide a more directly compatible legal substrate where they apply.

Fifth, the architecture leaves several surveillance concerns unaddressed by the design. The broader genomic surveillance concerns, such as (1) the 23andMe bankruptcy precedent, the Combined DNA Index System’s expansion through forensic genealogy [26], (2) the empirical finding that approximately two percent of a target population’s genetic information suffices to identify virtually any individual through familial matching [65], and (3) the Genetic Information Nondiscrimination Act gap in life, disability, and long-term care insurance [66,67], require legal rather than architectural remedies. The same is true for the nanotechnology and brain–computer-interface concerns that surveillance scholars project as the next frontier [20,68]. The Guardian Card addresses one tier of the surveillance problem; it does not pretend to address the others.

Finally, although this article argues for a patient-possessed architecture on both clinical and constitutional grounds, the design should be evaluated empirically against the established centralized health information exchange model on outcome measures. These include emergency department turnaround time, medication-error rates at admission, patient-reported access experience, and the differential outcomes for the vulnerable populations identified by Joshi et al., Johnson et al., and McCreedy et al. rather than on architectural appeal alone [3,7,8].

To support such an evaluation, Table 4 proposes concrete metrics across three domains—clinical effectiveness, patient-centered experience, and economic impact—against which a deployed program could be assessed relative to a centralized comparator.

## 11. Conclusions

A portable, patient-possessed health record is now technically feasible using off-the-shelf standards, using HL7 FHIR for data representation, the World Wide Web Consortium’s Verifiable Credentials Data Model and SMART Health Cards for cryptographic portability, and a hybrid QR/NFC carrier for physical access. The clinical case for such a record is well established. The structural case is newer but, considering the surveillance-critical literature now accumulating, at least as compelling [10,25,29]. Centralized clinical-data architectures, however well intentioned, become substrates for the function creep, third-party doctrine erosion, and surveillance-instrumentarian repackaging documented across two decades of empirical and constitutional scholarship. Patient-possessed architectures, designed with the surveillance threat model as a first-class constraint rather than as an afterthought, neutralize the architectural asymmetry that makes the centralized model structurally vulnerable to capture.

The Guardian Card is offered as one operational implementation of a patient-controlled medication-record architecture grounded in the constitutional analysis the surveillance-critical literature advances [20,29]. It will not, on its own, resolve the broader structural problems the surveillance critique identifies; the prescription requirement itself, the warrantless-access state laws, the HIPAA exceptions, and the genomic and nanotechnology frontiers remain to be addressed through constitutional and legislative work [69]. However, a deployable architecture that solves the care-coordination problem without producing the surveillance substrate is a meaningful structural improvement, and one that can be built today. Anchoring issuance and maintenance at the community pharmacy, where the Pharmacist eCare Plan already provides a potential FHIR-conformant care-plan substrate, and where the populations most exposed to centralized surveillance harm interact most frequently with the clinical system, offers the most pragmatic and clinically meaningful first deployment venue.

## Figures and Tables

**Table 1:** Selected national health data aggregation systems, by architecture, coordinating body, platform, and year of national implementation [38,39,40,41,42,43,44,45,46,47,48].

Country	Architecture	Coordinating Body	National Platform	Year [Ref.]
Denmark	Centralized (federated)	MedCom; Ministry of Health; Danish Regions	Sundhed.dk	2003 [38]
Estonia	Centralized	TEHIK; Estonian Health Insurance Fund	EHIS/Terviseportaal (X-Road)	2008 [39]
Finland	Centralized	Kela; Ministry of Social Affairs and Health	Kanta/My Kanta Pages	2010 [40]
United Kingdom	Centralized	NHS Digital/NHS England	Summary Care Record (SCR)	2010 [41]
Norway	Centralized	Norwegian Directorate of eHealth	Helsenorge.no	2011 [42]
Australia	Centralized	Australian Digital Health Agency (ADHA)	My Health Record (MHR)	2012 [43]
Sweden	Centralized (federated)	Inera/E-hälsomyndigheten	NPÖ (National Patient Overview)	2013 [44]
Austria	Centralized	ELGA GmbH	ELGA (Elektronische Gesundheitsakte)	2015 [45]
Germany	Centralized	Gematik GmbH	ePA (Elektronische Patientenakte)	2021 [46]
India	Federated/ Portable	National Health Authority (NHA)	ABHA/Ayushman Bharat Digital Mission	2021 [47]
France	Centralized	Agence du Numérique en Santé (ANS)	Mon Espace Santé	2022 [48]

Abbreviations: ABHA, Ayushman Bharat Health Account; ADHA, Australian Digital Health Agency; ANS, Agence du Numérique en Santé; EHIS, Estonian Health Information System; ELGA, Elektronische Gesundheitsakte; ePA, Elektronische Patientenakte; MHR, My Health Record; NHA, National Health Authority; NPÖ, Nationell Patientöversikt; SCR, Summary Care Record; TEHIK, Tervise ja Heaolu Infosüsteemide Keskus.

**Table 2:** Comparison of QR Code and Near-Field Communication as Access Modalities.

Feature	QR Code	NFC (Chip)
Per-unit cost	Effectively zero (printing only)	Approximately $1–$5 per card
Compatibility	Any smartphone camera	Modern NFC-enabled smartphones
Durability	Vulnerable to scratching and fading	Internal; highly durable
Surveillance risk	Can be photographed from a distance	Requires ~2 inch proximity
Offline operation	Static payload only	Static payload only

**Table 3:** SWOT analysis of the patient-controlled, patient-possessed medication-record architecture.

Strengths	Weaknesses
Patient holds the integrative artifact; per-event consent for non-emergency access; patient-visible audit log; no central database to subpoena or breach; reduced re-identification surface; offline emergency access	Static payload requires reissuance after clinical changes; Tier 2 consent depends on patient mobile access; issuance and key-management burden on institutions; not yet a balloted standard for general clinical content
**Opportunities**	**Threats**
Community-pharmacy issuance can reach surveillance-exposed populations; the Pharmacist eCare Plan provides a FHIR-conformant substrate; alignment with verifiable-credential and patient-access policy momentum; improved continuity across care transitions and incarceration	Coerced consent by employers, insurers, or partners; break-glass logs as a re-entry point for compelled access absent legal protection; digital-divide exclusion; dependence on legal reforms the architecture cannot supply on its own

**Table 4:** Proposed criteria for evaluating a deployed patient-controlled medication-record program.

Domain	Representative Metrics
Clinical effectiveness	Medication-discrepancy and reconciliation-error rates at care transitions; emergency-department time-to-information and turnaround; completeness of allergy and active-medication data at the point of care; adverse-drug-event rates
Patient-centered outcomes	Patient-reported control over disclosure; comprehension of and engagement with the audit log; access experience among surveillance-exposed populations; consent-flow completion and refusal patterns; willingness to carry and use the credential
Economic impact	Issuance and key-management cost per patient; cost per averted adverse drug event or duplicative test; effects on downstream utilization and readmissions; total cost of ownership versus centralized exchange participation

## Data Availability

No new data were created or analyzed in this study. Data sharing is not applicable to this article.
